# Falls in older people: comparing older and younger fallers in a developing country

**DOI:** 10.1007/s00068-017-0818-2

**Published:** 2017-07-17

**Authors:** R. R. Yogi, I. Sammy, J. F. Paul, P. Nunes, P. Robertson, V. Ramcharitar Maharaj

**Affiliations:** 1 Southwestern Regional Health Authority, San Fernando, Republic of Trinidad and Tobago; 2 School of Health and Related Research, The University of Sheffield, Sheffield, S1 4HD UK; 3 The University of the West Indies, St Augustine, Trinidad; 4 North Central Regional Health Authority, Champs Fleurs, Republic of Trinidad and Tobago

**Keywords:** Aged, Accidental falls, Wounds and injuries, Hospital emergency service

## Abstract

**Purpose:**

While falls are common in older people, causing significant mortality and morbidity, this phenomenon has not been extensively studied in the Caribbean. This study aimed to compare falls in older and younger people in this setting.

**Methods:**

We conducted a prospective observational study of older trauma patients in Trinidad, comparing older and younger patients sustaining falls.

**Results:**

1432 adult trauma patients were included (1141 aged 18–64 years and 291 aged 65 years and older). Older fallers were more likely to be female (66.7 vs 47.2%; *p* < 0.001), suffer from multiple pre-existing diseases (24.7 vs 2.4%; *p* < 0.001) and take multiple medications (16.1 vs 0.8%; *p* < 0.001). They also sustained more severe injuries and presented with higher acuity than younger fallers. Admission rates were higher among older fallers (29.9 vs 13.1%; *p* < 0.001).

**Conclusions:**

In our study, older patients who fell were a distinct group from younger falls victims, with unique demographic, clinical and injury related characteristics. Their increased risk of injury within the home, coupled with their propensity for more severe injuries made them a high risk patient group. More research is needed to better understand this patient group and plan specific preventive interventions.

**Electronic supplementary material:**

The online version of this article (doi:10.1007/s00068-017-0818-2) contains supplementary material, which is available to authorized users.

## Introduction

While falls are common in older people, they remain a poorly understood mechanism of injury in the developing world [1, 2]. Many authors have investigated falls in developed countries, but little research has been done on this phenomenon in the Caribbean [3, 4]. Research into trauma and ageing in the developing world suggests that, while there are similarities between patients in developed and developing countries, there are also significant differences. Using data from 2009, Naraynsingh et al. showed that older trauma patients in the developing world represented a smaller proportion of all trauma victims seen in the Emergency Department (ED), compared to the developed world [2]. In that study, 10% of all patients were aged 65 and older, compared to 14.1% of UK trauma patients being 75 and older in the same year in the UK [5]. In addition, 33.1% of all trauma in Naraynsingh’s was accounted for by falls, while in the UK study, falls made up 44.3% of all injuries. Data from other developed countries, including the Europe and Canada, have showed that falls account for between 39.7 and 45.5% of all injuries seen in the ED, and that the incidence of falls increases with age [6–9].

The population in the developing world is ageing rapidly, and trauma in older people is becoming a significant public health issue. By the year 2050, 1 in 5 people in the developing world will be 60 years or older [10]. In these countries, a better understanding of falls in older people would improve outcomes in this vulnerable age group. Given the ageing of the population in the developing world, and the increased risk of falls with increased age, it is likely that the prevalence of falls will increase with time in developing countries.

Naraynsingh et al. documented the differences between younger and older trauma patients in Trinidad and Tobago, but did not specifically look at falls as a mechanism of injury [2]. Trinidad and Tobago is an Commonwealth Caribbean state, of 1.3 million people, whose main economic activities are centred around petroleum production and manufacturing. Currently, approximately 12% of its population is 60 years, but this is set to increase to 20% by 2050 [11]. Given the lack of data on falls in Trinidad in particular and the developing world in general, we set out to compare falls in older and younger patients presenting to the Emergency Department in this developing country.

## Methods

A prospective study of adult patients (≥18 years) presenting with falls to the Emergency Department of the San Fernando General Hospital (a tertiary teaching hospital) was conducted over 4 months. The aim was to determine the demographic and clinical differences between older patients (aged ≥65 years) and younger patients (aged 18–64 years) with falls.

Data were collected using a data collection form designed for this study, which was completed by the staff working in the Emergency Department when the patient was admitted. The data collection forms were cross referenced with patients’ clinical notes by the lead researcher to complete any missing data.

Older and younger patients were compared in relation to demographic characteristics (age, gender and ethnicity); pre-existing medical status (pre-existing medical conditions and pre-injury medication); injury characteristics (injury pattern and severity) and outcome. All injuries were coded using ICD-10 codes, and abbreviated injury scores (AIS) were extrapolated from the ICD-10 codes for each injury. For ICD-10 codes that mapped to more than one AIS, the lowest matching AIS was used. Statistical analysis was performed using SPSS version 18.0. Categorical data were compared using Chi-Squared analysis, while Student’s *t* test for independent variables was used to compare continuous data.

Ethical approval was obtained from the research ethics committees of the Southwestern Regional Health Authority and the University of the West Indies, St Augustine; all data were anonymised at the point of collection. Informed consent was obtained from all individual participants included in the study.

## Results

1432 patients were included in this study: 1141 aged 18–64 years and 291 aged ≥65 years (Online Appendix 1). The mean age of the sample was 41.9 years (95% CI 40.9–42.8 years); 732 (51.1%) were female and 700 (48.9%) patients were male.

Table 1 summarises the demographic and clinical characteristics of younger and older patients admitted after a fall. Older fallers were more likely to be female. Of the older fallers, 72 (24.7%) had 3 or more pre-existing medical conditions, compared to 27 (2.4%) in the younger age group (*p* < 0.001). Polypharmacy (≥5 medications) was seen in 47 (16.1%) older patients versus 10 (0.8%) younger patients (*p* < 0.001).

**Table 1:** Demographic and clinical features of patients with falls, comparing younger and older patients

Demographic and clinical characteristics compared by age group				
	18–64 years (*n* = 1141)	≥65 years (*n* = 291)	Total (*n* = 1432)	*p* value
Mean age				
Age (95% CI)	34.7 (33.9–35.5)	70.3 (69.3–71.3)	41.9 (40.9–42.8)	
Sex				
Male [*n* (%)]	603 (52.8%)	97 (33.3%)	700 (48.9%)	<0.001
Female [*n* (%)]	538 (47.2%)	194 (66.7%)	732 (51.1%)	
Number of pre-existing medical conditions				
0 [*n* (%)]	854 (74.8%)	57 (19.6%)	911 (63.6%)	<0.001
1–2 [*n* (%)]	260 (22.8%)	161 (55.4%)	421 (29.4%)	
≥3 [*n* (%)]	27 (2.4%)	72 (24.7%)	99 (6.9%)	
Not recorded [*n* (%)]		1 (0.3%)	1 (0.1%)	
Number of drugs				
0 [*n* (%)]	853 (74.9%)	61 (20.9%)	914 (63.9%)	<0.001
1–4 [*n* (%)]	273 (23.9%)	182 (62.7%)	455 (31.8%)	
≥5 [*n* (%)]	10 (0.8%)	47 (16.1%)	57 (3.9%)	
Not recorded [*n* (%)]	5 (0.4%)	1 (0.3%)	6 (0.4%)	
Site of injury				
In the home [*n* (%)]	708 (62.1%)	251 (86.3%)	959 (67%)	<0.001
In the garden [*n* (%)]	44 (3.9%)	16 (5.5%)	60 (4.2%)	
In a public place [*n* (%)]	166 (14.5%)	22 (7.6%)	188 (13.1%)	
At work [*n* (%)]	116 (10.2%)	0 (0%)	116 (8.1%)	
Playing a sport [*n* (%)]	107 (9.4%)	2 (0.7%)	109 (7.6%)	
Mechanism of injury				
Fall from a height [*n* (%)]	187 (16.4%)	39 (13.4%)	226 (15.8%)	0.242
Fall from standing [*n* (%)]	954 (83.6%)	252 (86.6%)	1206 (84.2%)	
Falls from standing (causes)				
Slip/trip [*n* (%)]	774 (81.1%)	211 (83.7%)	985 (81.7%)	<0.001
Seizure [*n* (%)]	126 (13.2%)	10 (4.0%)	136 (11.3%)	
Syncope [*n* (%)]	33 (3.5%)	7 (2.8%)	40 (3.3%)	
Other [*n* (%)]	21 (2.2%)	24 (9.5%)	45 (3.7%)	
No. of injuries				
1 [*n* (%)]	956 (83.8%)	226 (77.7%)	1182 (82.5%)	0.009
≥2 [*n* (%)]	185 (16.2%)	65 (22%)	250 (17.4%)	
Patients with at least one injury				
AIS ≥ 3 [*n* (%)]	25 (2.2%)	23 (7.9%)	48 (3.4%)	0.001
Not recorded [*n* (%)]	4 (0.4%)		4 (0.3%)	
CTAS triage category				
1 (Life threatening) [*n* (%)]	1 (0.1%)	0 (0%)	1 (0.1%)	<0.001
2 (Emergent) [*n* (%)]	8 (0.7%)	6 (2.1%)	14 (1%)	
3 (Urgent) [*n* (%)]	200 (17.5%)	96 (33%)	296 (20.7%)	
4 (Less urgent) [*n* (%)]	932 (81.7%)	188 (64.6%)	1120 (78.2%)	
5 (Non-urgent) [*n* (%)]	0 (0%)	1 (0.3%)	1 (0.1%)	
Outcome				
Admission [*n* (%)]	150 (13.1%)	87 (29.9%)	237 (16.6%)	<0.001
Specialist referral [*n* (%)]	330 (28.9%)	81 (27.8%)	411 (28.7%)	
Discharge (without referral) [*n* (%)]	627 (55%)	118 (40.5%)	745 (52%)	
DAMA [*n* (%)]	34 (3%)	4 (1.4%)	38 (2.7%)	
Died [*n* (%)]	0 (0%)	1 (0.3%)	1 (0.1%)	

*DAMA* discharged against medical advice

The prevalence of falls from standing and falls from a height was not significantly different between age groups. However, in older patients suffering falls from standing, the underlying causes were significantly different to those in younger patients. Both groups reported a ‘slip/trip’ as the commonest underlying cause for their fall, but seizures were more common in younger people [126 (13.2%) vs 10 (4.0%)], and other causes (including chest pain, shortness of breath and pre-syncope) were more common in older people [24 (9.5%) vs 21 (2.2%)]. There were also significant differences in the site at which the fall occurred. Older patients were significantly more likely than younger patients to have fallen in the home [251 (86.3%) vs 708 (62.1%); *p* < 0.001], while younger patients were more likely to have fallen at work [116 (10.2%) vs 0(0%)], in a public place [166 (14.5%) vs 22 (7.6%)] or playing a sport [107 (9.4%) vs 2 (0.7%)] (*p* < 0.001).

Older patients were significantly more likely to sustain severe injuries; 23 (7.9%) older patients sustained at least one injury with an AIS ≥ 3, compared to 25 (2.2%) younger patients (*p* = 0.001). This was mainly due to older patients having more severe limb injuries: 18 (9.0%) older patients sustained limb injuries with an AIS ≥ 3, compared to 7 (0.9%) younger patients. There were no significant differences between older and younger patients in the proportion of serious injuries in other body regions.

Older patients were more likely to be assigned a more urgent triage acuity compared to younger patients (see Table 1). In the older cohort of patients, 102 (35.1%) were triaged to CTAS category 1, 2 or 3, compared to 209 (18.3%) younger patients (*p* < 0.001). However, most patients from both age groups were assigned less severe CTAS triage categories (CTAS level 4–5). Older patients were more likely to be admitted to hospital [87 (29.9%) vs 150 (13.1%)], while younger patients were more likely to be discharged [627 (55%) vs 118 (40.5%); *p* < 0.001]. Rates of referral for specialist care were similar in older and younger patients. There were very few deaths recorded in either age group.

## Discussion

Older patients with falls were a demographically and clinically distinct group from their younger counterparts, with more pre-existing medical conditions, more medications, higher triage acuity, more severe injuries and a greater likelihood of admission.

Our finding that older fallers are more likely to have pre-existing medical conditions and be on multiple medications has been observed by other authors, in the context of trauma in general. Wardle et al. [12] commented on the increase in comorbid conditions in older trauma patients in the UK and hypothesised a link between comorbidities and outcome. More recently, Grossman et al. demonstrated the incidence and impact of comorbidities on older trauma patients, while others have commented on the prevalence of certain medications (such as warfarin) in this cohort of patients [13, 14]. Sampalis et al., comparing older fallers with patients involved in motor vehicle collisions (MVCs), found that older fallers had more comorbidities and a higher Charlson Comorbidity Index than those involved in MVCs.

Previous research into falls in older people demonstrated its seriousness. Sterling et al. demonstrated that older patients with falls had higher injury severity and mortality than younger fallers, while Spaniolas et al., comparing ground level falls in older and younger adults found that the older cohort suffered more serious injuries and were more likely to have adverse outcomes, including death [3, 15]. Our study also demonstrated that older falls victims had a higher injury severity and triage acuity than younger fallers. However, the injury patterns (particularly head and chest injuries) reported in other studies were not seen in our patients. Spaniolas, Sterling and others have previously reported a high incidence of head and chest injuries in older fallers. In Sterling’s study, 47% of older fallers had head and neck injuries (compared to only 22% of younger fallers), and 23% had chest injuries (compared to 8% of younger fallers), while in Spaniolas’ study of older people with falls from standing, 9.7% sustained severe traumatic brain injuries [3, 15]. In contrast, our study did not show any significant differences between the age cohorts with regard to brain or chest injuries. In keeping with previous research, our study demonstrated a propensity to more severe limb injuries in older fallers, possibly due to the greater prevalence of osteoporosis. However, the prevalence of severe limb injuries of 9% seen in our study was lower than the prevalence noted in both Sterling’s study (which recorded a prevalence of combined pelvic and extremity injuries of 27%) and Spaniolas’ study (which recorded an incidence of limb fractures of 46.3%) [3, 15]. The finding in our study that older patients were more likely to be admitted to hospital also concurs with other authors, and may be related to the severity of their injuries or the impact of their pre-existing medical status [16].

## Limitations

This is the only study we know of that has compared falls in older and younger adults in a developing world setting. While findings of this study will be useful in planning services for older trauma patients in the developing world, there were a few limitations. It was a single centre study, with a risk of selection bias. However, the San Fernando Hospital serves approximately half of the population of Trinidad and Tobago, and the hospital’s patients are broadly representative of the general population [2]. The low AIS scores in this study may be because the lowest possible score was used when converting ICD-10 codes to AIS. However, the overall injury severity in our study was likely to be substantially lower than in other studies using trauma registries, as registries only include patients with more serious injuries. The mortality rate for this study was low, partly due to the liberal inclusion criteria, but also because only in-hospital mortality was measured—the study did not include trauma victims who died before reaching hospital or those who died after discharge.

## Conclusion

Older people who suffer falls are a distinct group of patients, and their characteristics in the Trinidad population are different to older falls patients in the developed world. A prospective, multicentre study is planned across the three tertiary hospitals in Trinidad, which would compare older and younger fallers over the entire island. We believe that the larger sample size and wider geographic sampling area would provide a more accurate reflection of the differences between older and younger fallers in Trinidad. We are in the process of establishing a trauma registry across all hospitals in Trinidad and Tobago, which will provide ongoing data on trauma in Trinidad and monitor trends in the epidemiology, treatment and outcome of this global epidemic. The ongoing collection of data on trauma patients will be critical in understanding the characteristics of trauma patients in Trinidad, while also providing data on the effectiveness of treatment provided. These data will help us to further improve our trauma services using a locally developed, evidence based approach.

## Electronic supplementary material

Below is the link to the electronic supplementary material.

Supplementary material 1 (DOCX 26 kb)

## References

[1] Yeung JH, Chang AL, Ho W, So FL, Graham CA, Cheng B (2008). High risk trauma in older adults in Hong Kong: a multicentre study. Injury.

[2] Naraynsingh R, Sammy I, Paul JF, Nunes P (2015). Trauma in the elderly in Trinidad and Tobago: a cross-sectional study. Eur J Emerg Med..

[3] Sterling DA, O’Connor JA, Bonadies J (2001). Geriatric falls: injury severity is high and disproportionate to mechanism. J Trauma..

[4] Sampalis JS, Nathanson R, Vaillancourt J, Nikolis A, Liberman M, Angelopoulos J (2009). Assessment of mortality in older trauma patients sustaining injuries from falls or motor vehicle collisions treated in regional level I Trauma Centers. Ann Surg.

[5] Kehoe A, Smith JE, Edwards A, Yates D, Lecky F (2015). The changing face of major trauma in the UK. Emerg Med J..

[6] Kehoe AD, Smith JE, Lecky F, Yates D (2014). Presenting gcs in elderly patients with isolated traumatic brain injury is higher than in younger adults. Emerg Med J..

[7] Belzunegui T, Gradin C, Fortun M, Cabodevilla A, Barbachano A, Sanz JA (2013). Major trauma registry of Navarre (Spain): the accuracy of different survival prediction models. Am J Emerg Med.

[8] Curtis KA, Mitchell RJ, Chong SS, Balogh ZJ, Reed DJ, Clark PT (2012). Injury trends and mortality in adult patients with major trauma in New South Wales. Med J Aust.

[9] Laupland KB, Kortbeek JB, Findlay C, Hameed SM (2005). A population-based assessment of major trauma in a large Canadian region. Am J Surg..

[10] World Population Ageing 1950–2050: United Nations, Department of Economic and Social Affairs, Population Division; 2002. http://www.un.org/esa/population/publications/worldageing19502050/Executivesummary_English.pdf. Accessed 5 June 2016.

[11] World Population Aging 2013 New York: United Nations, Department of Economic and Social Affairs, Population Division; 2013. http://www.un.org/en/development/desa/population/publications/pdf/ageing/WorldPopulationAgeing2013.pdf. Accessed 8 June 2016.

[12] Wardle T (1999). Co-morbid factors in trauma patients. Br Med Bull.

[13] Grossman MD, Miller D, Scaff DW, Arcona S (2002). When is an elder old? Effect of preexisting conditions on mortality in geriatric trauma. J Trauma.

[14] Batchelor JS, Grayson A (2012). A meta-analysis to determine the effect of anticoagulation on mortality in patients with blunt head trauma. Br J Neurosurg.

[15] Spaniolas K, Cheng J, Gestring M, Sangosanya A, Stassen N, Bankey P (2010). Ground level falls are associated with significant mortality in elderly patients. J Trauma.

[16] Naghavi M, Wang H, Lozano R, Davis A, Liang X, Zhou M (2015). Global, regional, and national age-sex specific all-cause and cause-specific mortality for 240 causes of death, 1990–2013: a systematic analysis for the Global Burden of Disease Study 2013. Lancet..

