# Supplementary material for: Falls in older people: comparing older and younger fallers in a developing country
Source: Eur J Trauma Emerg Surg. 2017 Jul 17;44(4):567–71. doi: 10.1007/s00068-017-0818-2 (PMC6096617; doi:10.1007/s00068-017-0818-2)
Supplement: Supplementary file 1 — Supplementary material 1 (DOCX 26 kb) [file 68_2017_818_MOESM1_ESM.docx]

**Appendix1: The recruitment flow diagram for the study.**
